# Supplementary material for: Generation of Wheat Near-Isogenic Lines Overexpressing 1Bx7 Glutenin with Increased Protein Contents and SDS-Sedimentation Values
Source: Plants (Basel). 2023 Mar 9;12(6):1244. doi: 10.3390/plants12061244 (PMC10051937; doi:10.3390/plants12061244)
Supplement: Supplementary file 1 [file plants-12-01244-s001.zip › plants-2188697-supplementary.pdf]

Supplementary Data

Generation of Wheat Near-Isogenic Lines Overexpressing 1Bx7 Glutenin with Increased Protein Contents and SDS-Sedimentation Values

Myoung-Hui Lee, Changhyun Choi, Kyeong-Hoon Kim, Jae-Han Son, Go-Eun Lee, Jun-Yong Choi, Chon-Sik Kang, Jiyoung Shon, Jong-Min Ko and Kyeong-Min Kim *

Wheat Research Team, National Institute of Crop Science, Rural Development Administration, Wanju 55365, Republic of Korea

* Correspondence: raiders87@korea.kr; Tel.: +82-63-238-5458

Table S1. Primers used for the selection of Bx7 and Bx7^{OE} cultivars.

Primer name	5–3’	Product size	Region	Reference
MAR-F<br>MAR-R	CTCAGCATGCAAACATGCAGC<br>CTGAAACCTTTGGCCAGTCATGTC	520/563 bp	43 bp indel	Butow et al. 2004
TaBAC1215C06-F517<br>TaBAC1215C06-R964	ACGTGTCCAAGCTTTGGTTC<br>GATTGGTGGGTGGATACAGG	447 bp	Left junction	Ragupathy et al. 2008
TaBAC1215C06-F24671<br>TaBAC1215C06-R25515	CCACTTCCAAGGTGGGACTA<br>TGCCAACACAAAAGAAGCTG	844 bp	Right junction	Ragupathy et al. 2008

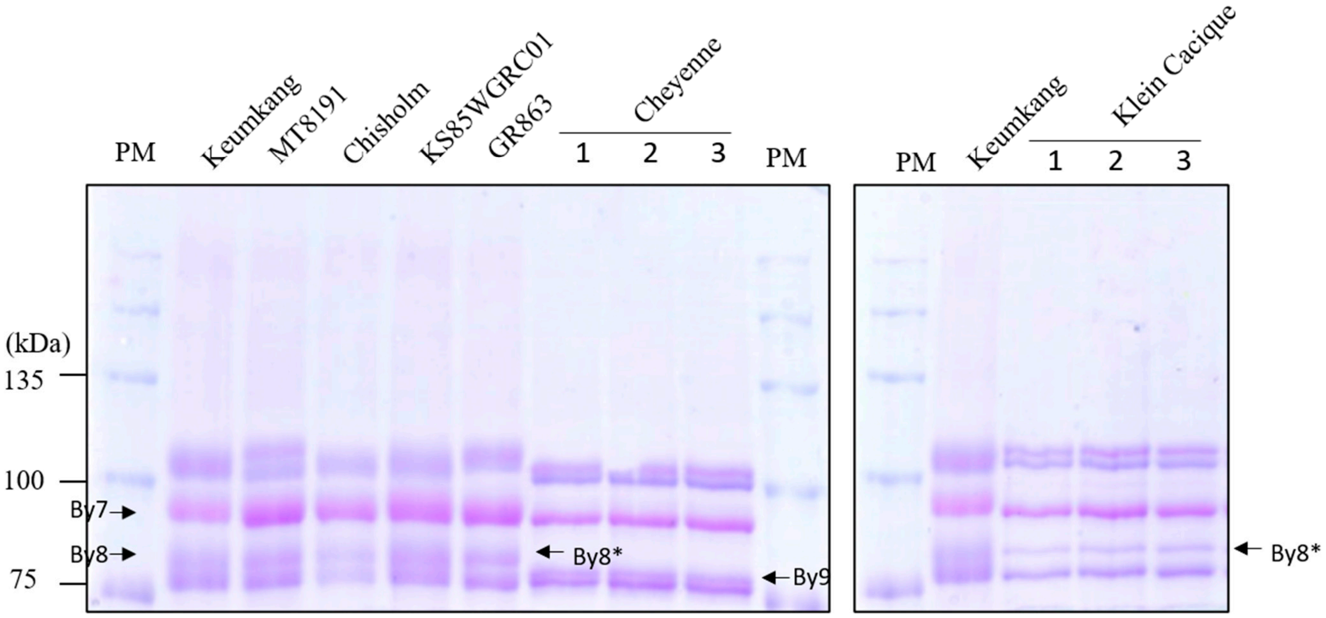

Figure S1. Analysis of HMW-GS fractions of seven wheat cultivars using 7% SDS-PAGE gel. PM, protein marker. Seven µg (Keumkang, MT8191, Chisholm, KS85WGRC101, and GR863) or 10 µg protein (Cheyenne and Klein Cacique) were separated on a 7% SDS-PAGE gels. The original gels are presented in Supplementary Figure S7.

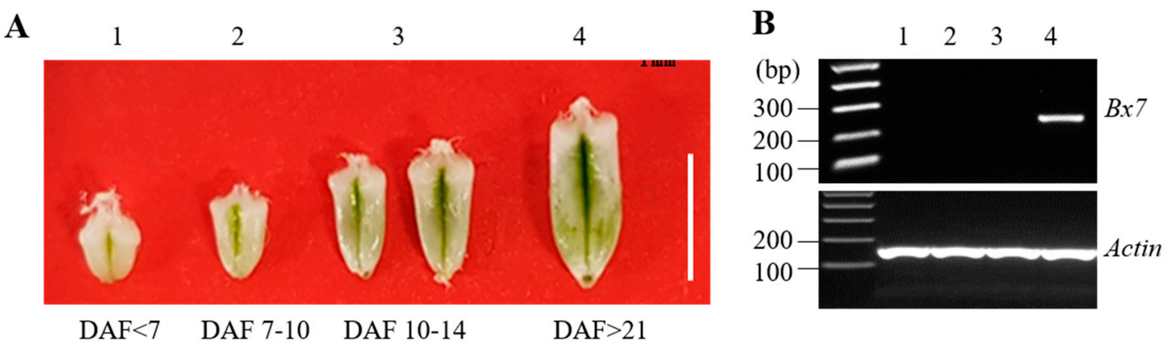

Figure S2. Analysis of *1Bx7* expression in immature seeds of Keumkang at designated days after flowering. A, Immature wheat seeds isolated 7–21 d post flowering. B, Reverse transcription PCR of *1Bx7*. *Actin* was used as a control. Scale bar, 0.5 cm. DAF, days after flowering. The original gel is presented in Supplementary Figure S8.

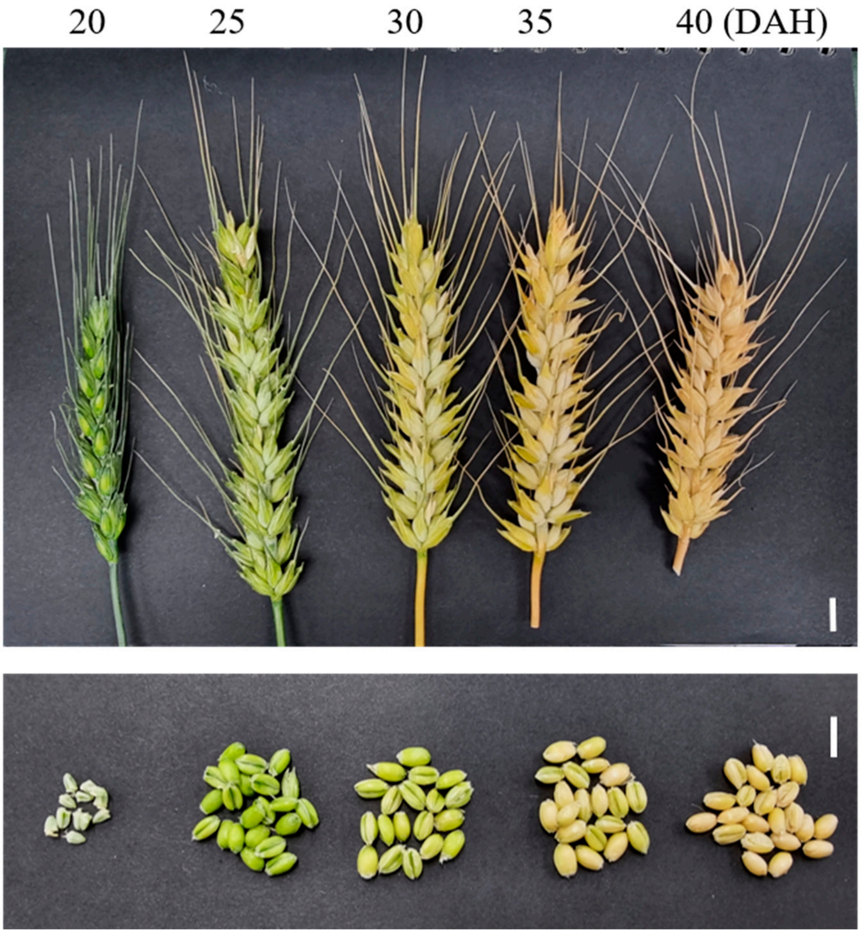

Figure S3. Maturation stages Keumkang spikes and seeds. DAH, days after heading. Scale bar, 1 cm. The original blots are presented in Supplementary Figure S5.

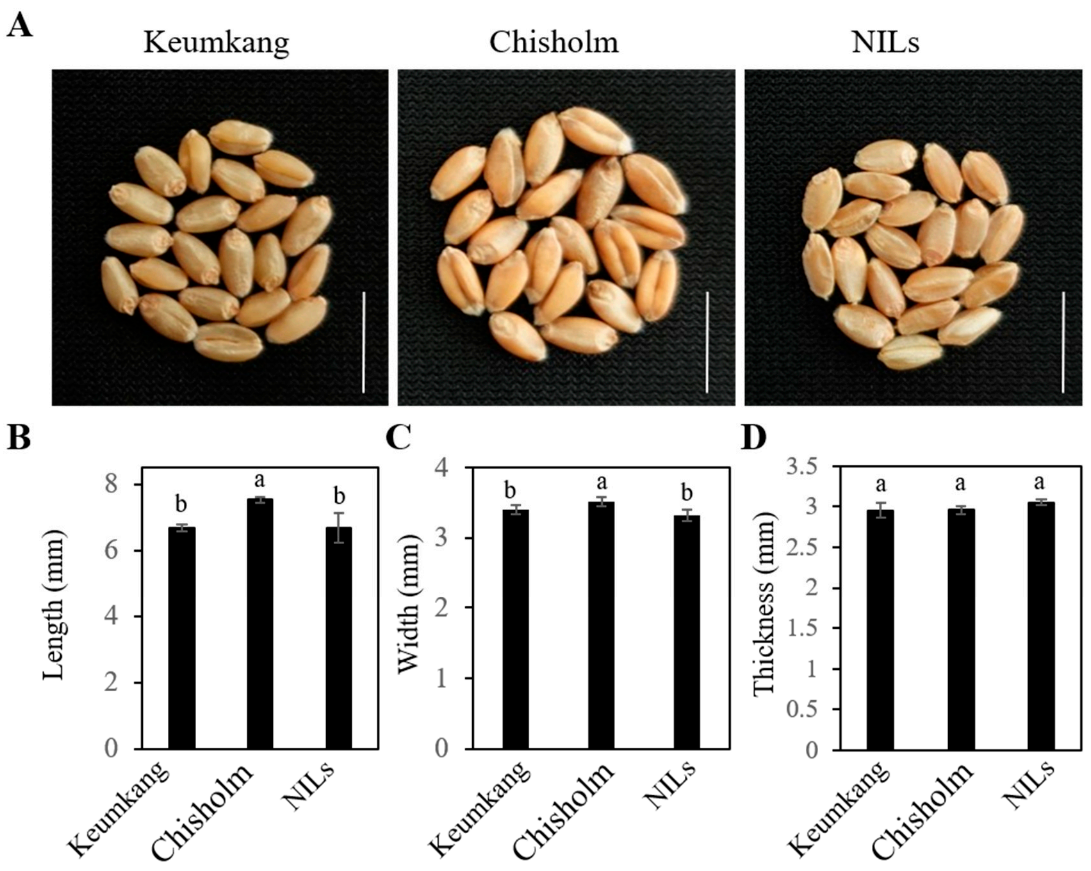

Figure S4. Grain characteristics of Keumkang, Chisholm, and NILs. A, Grain images. B-D, Seed length (B), width (C), thickness (D). Different letters on the bar indicate significant differences between treatments by Duncan's multiple comparison test, $p < 0.05$. Scale bar, 1 cm.

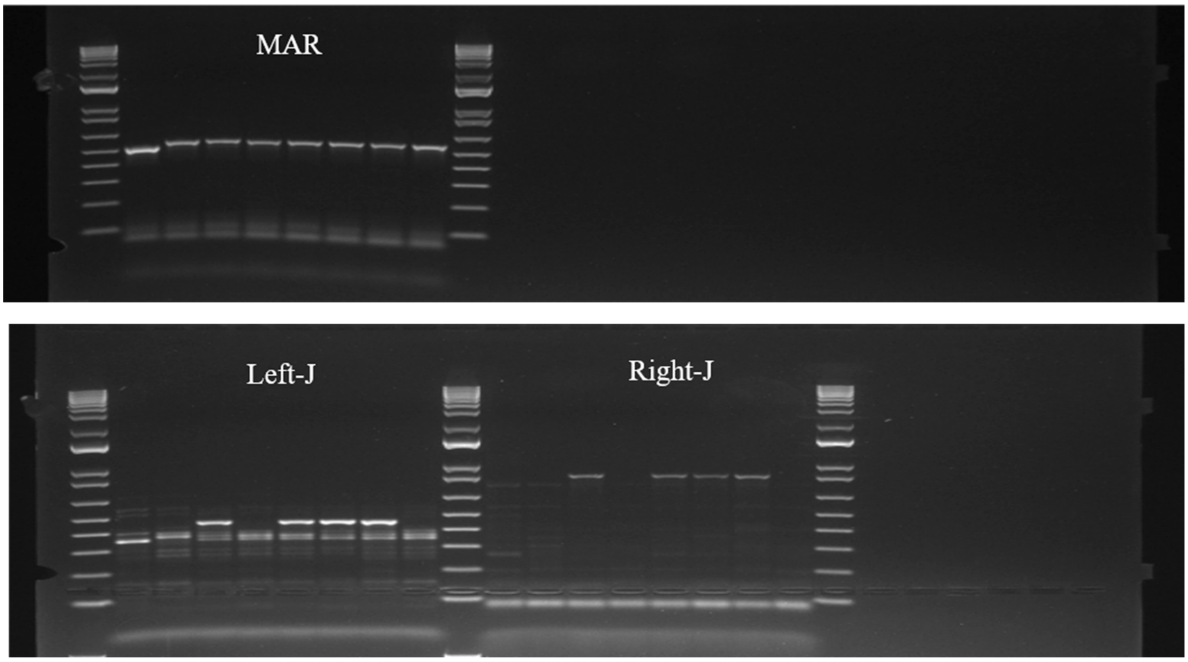

Figure S5. Original gel images shown in Figure 1.

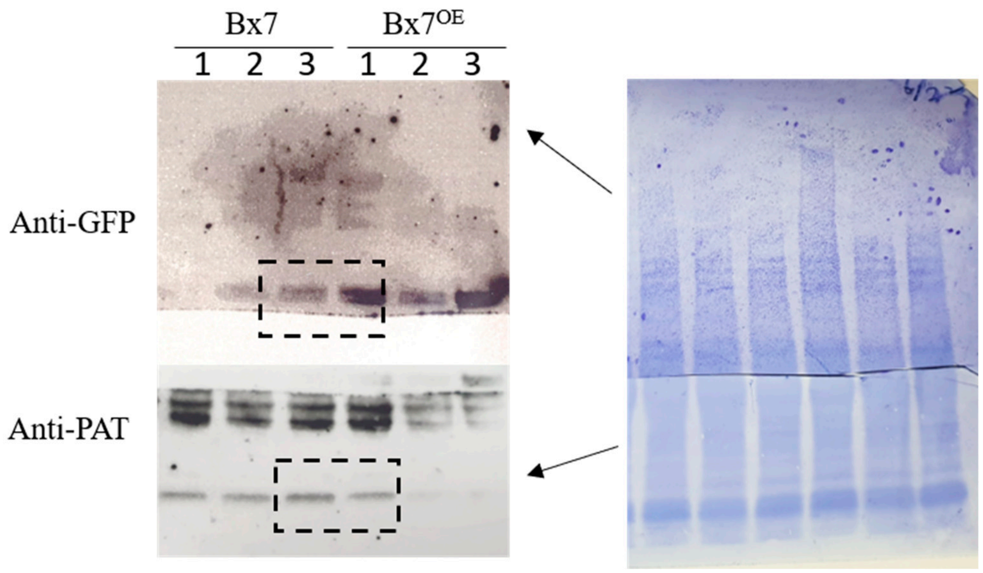

Figure S6. Original blots images shown in Figure S3B.

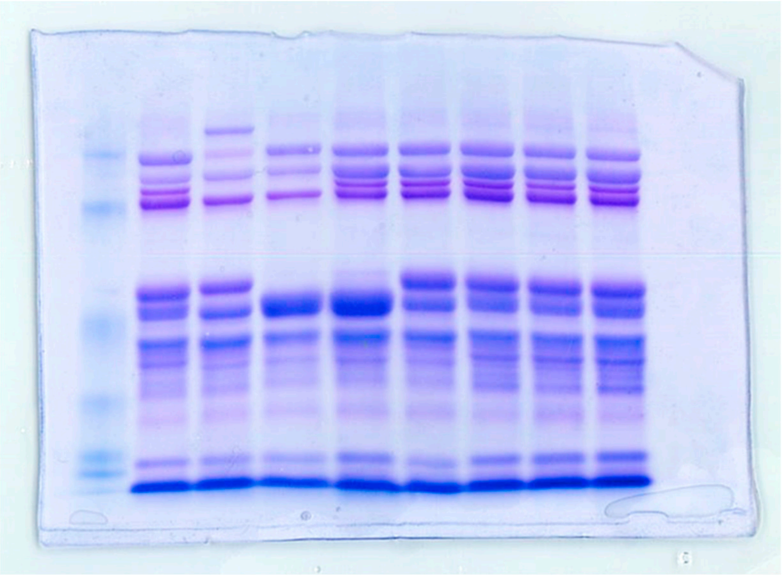

Figure S7. Original gel image shown in Figure 4.

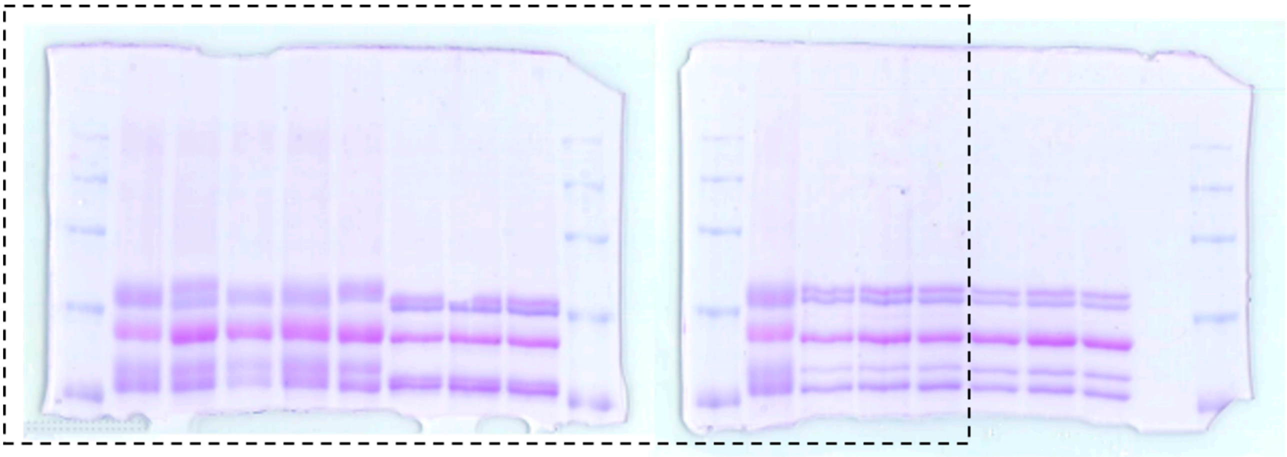

Figure S8. Original gel images shown in Figure S1.

Bx7

Actin

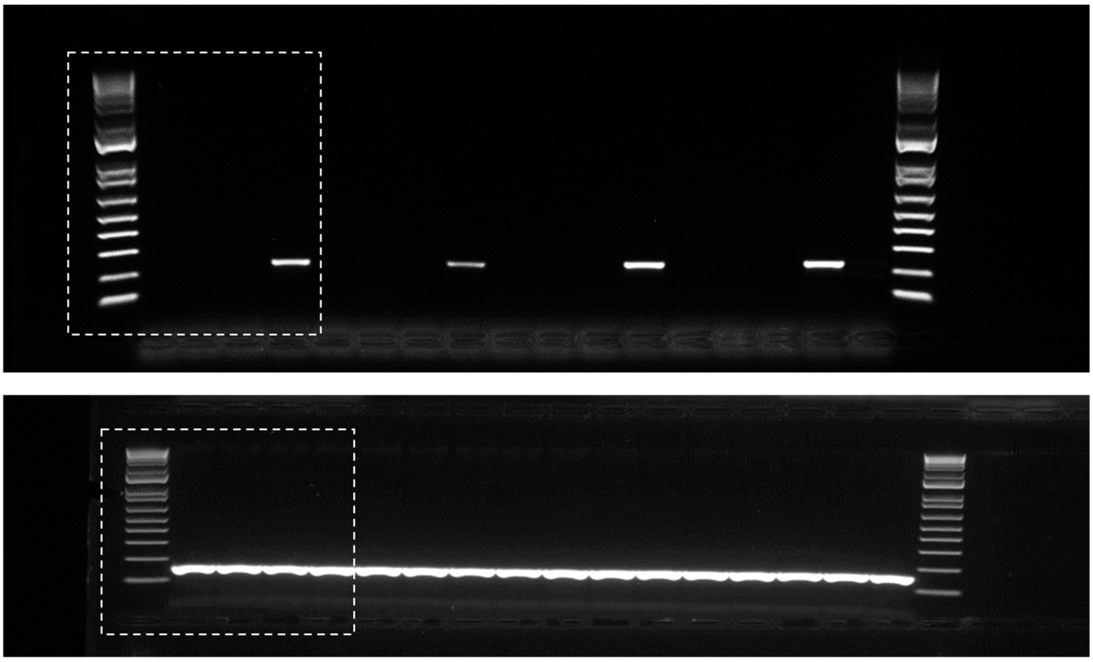

Figure S9. Original gel image shown in Figure S2B.
